# Causality Detection Methods Applied to the Investigation of Malaria Epidemics

**DOI:** 10.3390/e21080784

**Published:** 2019-08-11

**Authors:** Teddy Craciunescu, Andrea Murari, Michela Gelfusa

**Affiliations:** 1 National Institute for Laser, Plasma and Radiation Physics, RO-077125 Magurele-Bucharest, Romania; 2 Consorzio RFX (CNR, ENEA, INFN, Universita’ di Padova, Acciaierie Venete SpA), 35127 Padova, Italy; 3 Department of Industrial Engineering, University of Rome Tor Vergata, 00133 Rome, Italy

**Keywords:** dynamic system coupling, Granger causality, transfer entropy, recurrence plots, causal decomposition, cross-visibility graphs, malaria epidemics

## Abstract

Malaria, a disease with major health and socio-economic impacts, is driven by multiple factors, including a complex interaction with various climatic variables. In this paper, five methods developed for inferring causal relations between dynamic processes based on the information encapsulated in time series are applied on cases previously studied in literature by means of statistical methods. The causality detection techniques investigated in the paper are: a version of the kernel Granger causality, transfer entropy, recurrence plot, causal decomposition and complex networks. The methods provide coherent results giving a quite good confidence in the conclusions.

## 1. Introduction

Malaria is one of the deadliest diseases, and it still has a huge social, economic, and health impact. The disease has been declared to be endemic in 109 countries, and around one million deaths are reported annually, affecting mainly children under 5 years. Malaria is present mainly in the tropical regions, and there are concerns that global warming will increase its burden [1]. Certain studies (see, e.g., [2]) predict that a significantly expanded area of the globe could become vulnerable under climate change, while others predict only a geographic shift, but this shift could lead to an increased number of exposed people [3,4]. According to the Intergovernmental Panel on Climate Change (IPCC), the risk will become significant when the global temperature anomaly exceeds 1 °C, and dramatic when above 2 °C [5].

Therefore, climate-driven malaria epidemic studies represent a hot research topic. Even though a lot of effort has been devoted to understanding the onset and evolution of the disease, many aspects remain elusive. The main reason for this is the complexity of the ensemble of processes related to malaria epidemics. The agent causing the disease is Plasmodium, a eukaryotic protozoan, which requires a transmitting vector—the Anopheles mosquitos, and mediator hosts—the humans and the mosquitos, to complete its life cycle. From the five existent Plasmodium species that infect humans, two—P. vivax and P. falciparum—are responsible for almost all malaria deaths. Plasmodium enters the human body by means of a mosquito bite and reproduces by schizogony (asexual clonal expansion of many daughter spores) first in liver and afterwards in red blood cells. A subset of the offspring created in the red blood cells sexually differentiate and, with the occasion of another mosquito bite, they succeed to reproduce in the mosquito midgut. P. falciparum and P. vivax also have a dormant liver form, which may lead to reinfection years later [6]. For a detailed description of this complex process, and for an historical review of the disease, the reader is referred to [7]. The aspect most relevant for the studies presented in this paper is the fact that survival, reproduction, distribution of vectors and hosts, their living environment, and also the transmission process, are all influenced by climate conditions.

Malaria incidence has been linked mainly to the evolution of rainfall, temperature, humidity, density and health status of the vegetation (by means of the normalized difference vegetation index (NDVI) [8]. NDVI quantifies the vegetation fluctuations by measuring the difference between near-infrared radiation and visible light. Healthy and dense vegetation strongly absorbs the visible light received from the sun, while it strongly reflects near-infrared light. A positive influence of the El Niño Southern Oscillation (ENSO), measured by the Southern Oscillation Index (SOI), has also been reported [9]. However, the relation between malaria incidence and various climatic factors is not easy to determine, as it mixes with other factors, which are sometimes difficult to quantify, like, for example, socio-economic conditions, population mobility and growth insecticide resistance, effective treatment measure, and land use changes [10].

The relationship between malaria epidemics and climatic factors has been studied mainly using two approaches. On the one hand, a wide variety of process-based mathematical models, describing the non-linear dynamics of malaria transmission, have been proposed. An excellent historical overview of this family of methods is presented in [7]. On the other hand, various studies have been based on different methods for inferring information directly from time series, without modelling the underlying processes. Most of these approaches rely on techniques like rank and cross correlation, various kinds of regression, vector auto-regression (VAR), auto-regressive integrated moving average (ARIMA), time series density analysis, and wavelet analysis [11]. The approaches based on methods developed for revealing the coupling between time series have been less extensively exploited. Only a few applications of the Granger causality (see, e.g., [12,13,14]) have been reported.

This paper intends to bring to bear a series of methods from this family of time series analysis for causality and to show their efficacy in exploring the linkage between malaria epidemics and climate factors. The methods used are the kernel Granger causality [15,16,17], transfer entropy [18,19], recurrence plots [20], causal decomposition [21] and complex networks [22]. The deployment of multiple methods based on independent numerical procedures and providing coherent results, increase significantly the confidence in the conclusions. Moreover, the comparison of the various techniques makes it possible to estimate the confidence intervals in their estimates, a prohibitive task if only a single approach is utilized. With regard to the structure of the paper, the next section briefly describes the methods. The following section is devoted to the evaluation of the method’s performances. Several conclusions are drawn in last section of the paper.

## 2. Methods

### 2.1. Kernel Granger Causality (KGC)

The Granger causality method [15] is one major approach to accounting for causality between two time series. It formalizes a concept of causality initially proposed by Wiener [23], which is based on predictive capability: the cause must precede and enable prediction of the effect. The original Granger causality assumes linear dynamics. Because in real applications the interactions between dynamic systems are inherently nonlinear, various classes of non-linear Granger-based methods have been proposed. One of the most recent and powerful is briefly overviewed in the rest of this subsection.

Let $X={\left\{x_{i}\right\}}_{i=1,N}$ and $Y={\left\{y\right\}}_{i=1,N}$ be two stationary time series measured simultaneously. Y is said to Granger cause X if the prediction of X at the current time can be improved by incorporating past information regarding Y. The method hinges on linear prediction theory and uses an auto-regressive (AR) model. Therefore, the current value x, y of X and Y, respectively, can be expressed as a function of their past m values:

(1) $$\begin{array}{c} x=V_{1}\cdot X\\ y=V_{2}\cdot X \end{array}$$

with a prediction error:

(2) $$\begin{array}{c} \varepsilon_{x}=x-V_{1}\cdot X\\ \varepsilon_{y}=y-V_{2}\cdot X \end{array}$$

and where $V_{1}$ and $V_{2}$ are estimated by a least squares fit.

When the two time series are in a causal relationship, Equation (1) can be reformulated in order to include information about the other time series:

(3) $$\begin{array}{c} x=W_{11}\cdot X+W_{12}\cdot Y\\ y=W_{21}\cdot X+W_{22}\cdot Y \end{array}$$

where the elements of the matrix W are estimated again by a least squares technique. The prediction errors are:

(4) $$\begin{array}{c} \varepsilon_{xy}=x-W_{11}\cdot X-W_{12}Y\\ \varepsilon_{yx}=y-W_{21}\cdot X-W_{22}Y \end{array}$$

If the prediction error $\varepsilon_{xy}$, obtained by incorporating the past values of Y, is significantly smaller than $\varepsilon_{x}$, it means that Y has a causal influence on X. The comparison between the two variances can be performed by means of a statistical test like, e.g., the F-test. The strength of the causal interaction is defined by the relation:

(5) $$\delta =1-\frac{<\varepsilon_{xy}{}^{2}>}{<\varepsilon_{x}{}^{2}>}$$

where <·> means averaging over N. Similarly, if $\varepsilon_{yx}$ is significantly smaller than $\varepsilon_{y}$, X has a causal influence on X.

A geometrical view of Granger causality was introduced in [16,17]. It starts from the notations:

(6) $$\begin{array}{c} X_{i}^{*}={\left(x_{i},\ldots ,x_{i+m-1}\right)}^{⊺}\\ Y_{i}^{*}={\left(y_{i},\ldots ,y_{i+m-1}\right)}^{⊺}\\ x_{i}^{*}=x_{i+m} \end{array}$$

where i=1,…,N. The quantities in Equation (6) can be treated as realizations of the stochastic variables X, Y, and x. Further, $X^{*}$ is the m×N matrix whose columns are the vectors $X_{i}^{*}$ and $x^{*}={\left(x_{1}^{*},\ldots ,x_{N}^{*}\right)}^{⊺}$. The value of the regression of $x^{*}$ versus $X^{*}$ is given by the relation:

(7) $$\tilde{x_{i}^{*}}=\overset{m}{\underset{j=1}{\sum }}A_{j}x_{i+m-j}$$

If we denote by $H\subseteq R^{N}$ the range of the matrix $K=X^{*⊺}X^{*}$, then $\tilde{x^{*}}$ can be interpreted as the projection of $x^{*}$ on H, $\tilde{x^{*}}=Px^{*}$ by means of the projector:

(8) $$P=\overset{m}{\underset{i=1}{\sum }}v_{i}v_{i}^{T}$$

where $\left\{v_{i}\right\}$ are the eigenvectors of K.

When considering both $X^{*}$ and $Y^{*}$, it is possible to construct the 2m×N matrix $Z^{*}$ with the vectors ${\left(X_{i}^{*⊺},\;Y_{i}^{*⊺}\right)}^{⊺}$ as columns. Similarly to Equation (7), the linear regression process leads to:

(9) $$\tilde{x^{*\prime }}=P^{\prime }x^{*}$$

where $P^{\prime }$ is the projection operator on the 2m-domensional space $H^{\prime }\subseteq R^{N}$ equal to the range of the matrix $K^{\prime }=Z^{*⊺}Z^{*}$.

$H^{\prime }$ can be decomposed in the form $H^{\prime }=H⊕H^{⊥}$, where $H^{⊥}$ links with the additional features related to the inclusion of {y} variables. $H^{⊥}$ is of the range of the matrix $\tilde{K}=K^{\prime }-PK^{\prime }-K^{\prime }P+PK^{\prime }P$, so the non-zero eigenvectors $\left\{t_{1,\;\ldots ,\;}t_{m}\right\}$ of $H^{⊥}$ can be used to construct an orthonormal base. A related projection operator $P^{⊥}$ can be introduced. Following the same steps, the causality index can be retrieved (see [3,4] for details):

(10) $$\delta \left(Y^{*}\to X^{*}\right)=\overset{m}{\underset{i=1}{\sum }}r_{i}^{2}$$

where $r_{i}$ is the Pearson correlation coefficient of the residual $r=x^{*}-\tilde{x^{*}}$ and the eigenvectors {$t_{i}\}$ of $\tilde{K}$.

The main advantage of this approach is the generalization to an arbitrary degree of nonlinearity by means of kernels $G\left(X,X^{\prime }\right)=\sum_{i}\lambda_{i}\Phi_{a}\left(X\right)\Phi_{a}\left(X^{\prime }\right)$ [16], which allow the mapping of variables in a higher dimensional space where, hopefully, the linear analysis can be applied. According to [16,17], the above-defined $K=X^{*⊺}X^{*}$ and $K^{\prime }=Z^{*⊺}Z^{*}$ should be replaced with Gram matrices with elements given by $K_{ij}=G\left(X_{i}^{*},X_{j}^{*}\;\right)$ and $K_{ij}^{\prime }=G\left(Z_{i}^{*},Z_{j}^{*}\;\right)$.

In the present approach we have used polynomial kernels:

(11) $$G\left(X,X^{\prime }\right)={\left(1+X^{⊺}X^{\prime }\right)}^{p}$$

where p is the polynomial order.

### 2.2. Transfer Entropy (TE)

Transfer Entropy (TE) was introduced by Schreiber [18] as a tool for measuring the exchange of information between joint processes. TE incorporate the flow of time by means of the conditional probabilities that describe the evolution of the system from its past states to its present state. Another advantage consists of its independence from any assumption about the interaction of the dynamical systems. Thanks to its inherent asymmetry, TE can provide information on the directionality of the causal influence. A close connection with Granger causality has been demonstrated [24].

Considering M processes $X_{M}$ with the corresponding time series ${\left\{x_{mn}\right\}}_{m=1,M;\;n=1,N}$, the state $x_{in}$ is visited by $X_{i}$ at time n with the associated probability $p(x_{in)}.$ It is assumed that this state is determined by k past states, which are collected in an embedding vector $x_{i,n}^{\left(k\right)}$ with the associated joint probability $p\left(x_{i,n}^{\left(k\right)}\right)$. The dynamics of the process is described by mean of the conditional probabilities $p(x_{in}|x_{i,n}^{\left(k\right)})$, which quantify the probability that the system $X_{i}$ is in the state $x_{in}$ when the preceding states are those collected in the embedding vector. The transition to a new state is quantified by the conditional entropy:

(12) $$H\left(x_{in}|x_{i,n}^{\left(k\right)}\right)=-\sum_{n}p\left(x_{in},x_{i,n}^{\left(k\right)}\right)\ln\left[p\left(x_{in}|x_{i,n}^{\left(k\right)}\right)\right]=\;H\left(x_{in},x_{i,n}^{\left(k\right)}\right)-H\left(x_{i,n}^{\left(k\right)}\right)$$

The assessment of causality between two processes $X_{i}$ and $X_{j}$ is grounded on the Granger principle. The entropy of $X_{i}$ conditioned on its own past and the past of all processes except $X_{j}$ is compared with the entropy conditioned by the past of all processes. A multivariate embedding $X^{K}=\left(x_{i,n}^{\left(k_{1}\right)},\ldots ,x_{i,n}^{\left(k_{M}\right)}\right)$ must be constructed. The coupling strength between the two processes is measured by the index:

(13) $$C_{j\to i}=1-\frac{H\left(x_{in}|X^{K}\right)}{H\left(x_{in}|X^{K_{j}}\right)}$$

which varies in the interval [0,1]. A uniform scheme for the construction of the embedding vector may lead to overfitting and false causalities [16,17]. Therefore, a non-uniform embedding scheme, whereby embedding vectors are built progressively on the basis of a minimization criterion applied to the entropy of the present state of the system conditioned to its past states [19], has been used. The scheme builds the conditioning vector V sequentially by selecting the time series terms which minimize the conditional entropy. At the beginning, V is an empty vector, and afterwards, at each step j, past terms of the investigated time series Y or also past terms of the potentially coupled time series X:

(14) $${\left\{X\right\}}_{j}{\left\{Y\right\}}_{j}\;,\;j=\;\left\{\left(n-\tau \right),\;\ldots ,\left(n-L\tau \right)\right\}$$

are added sequentially. L is the size of the initial set, and τ is a time delay which allows the selection of non-subsequent terms of the time series. As shown in [25], τ can be selected so that the decorrelation time of the time series [26] is below 1/e. All the candidate terms are tested by computing the entropy of Y conditioned to V, and the term which provides the minimum entropy is selected. The procedure continues until a minimum value is found among the selected candidates. For assessing the causality between X and Y, the procedure is applied two times: (i) first, $V_{1}$ contains only samples from Y; and then (ii) $V_{2}$ also includes terms from X, in order to allow the estimation of the index given by the relation (13). According to [25], the normalized series can be represented over Q quantization levels, enabling the computation of the entropies by approximating the probabilities with the occurrence frequency in each level Q. This approximation is reasonable if the number of quantization levels is correlated with the length of the time series. A relation for determining Q has been proposed in [27]:

(15) $$N\approx Q^{k}$$

### 2.3. Recurrence Plots (RP)

Recurrence plots (RP) were introduced as a 2-dimensional representation of the recurrence properties for systems evolving in an *m*-dimensional phase space [28]. The representation is a square matrix, with ones for the times at which a phase space trajectory visits roughly the same area in the phase space, and zeros otherwise:

(16) $$R_{ij}=\Theta \left(\varepsilon -\|\vec{x_{i}}-\vec{x_{j}}\|\right),\;\;\;\;i,j\;=\;1,\ldots ,N$$

where: $\vec{x_{i}}$_stands for the point in phase space at which the system is situated at time i, and ε is a predefined threshold. As Θ(x) is the Heaviside function, the matrix consists of the values 1 and 0 only. A norm should be defined in Equation (14). The Euclidean distance is a common choice. When the data is affected by significant noise, a metric based on the use of the Geodesic distance could improve the results. If the noise presents a Gaussian distribution, the measurements can be considered not as punctual values in Euclidean space, but as Gaussian distributions. Therefore, the distance between Gaussians is an appropriate choice. In particular, the Geodesic distance on Gaussian manifolds is a natural choice [29]. Special attention must be paid to the value of the threshold ε. Although there is no general rule for the estimation of ε, the noise level of the time series plays an important role in its selection. Usually, ε is chosen to be a percentage of the diameter of the reconstructed trajectory in the phase space not greater than 10% [30,31]. The ‘false neighbours’ method can be used for determining the embedding dimension of the phase space [32].

The graphical representation of RP, encoding 1 as black and 0 as white, depicts the collection of pairs of times at which the trajectory returns sufficiently close the same place. It is used to display recurring patterns and to investigate nonstationary patterns [28]. RPs preserve all the topologically relevant phase space information of the system, and therefore, a time series can be reconstructed from its RP up to a rescaling of its probability distribution function [33].

RPs can be assimilated to complex networks (see Section 2.5) by identifying the RP matrix with the adjacency matrix. This straightforward idea ensures the preservation of many local properties of the dynamical system observed by means of the time series [34].

Multivariate extensions of recurrence plots have been developed. The most relevant for the applications described in this paper are the joint recurrence plots (JRP) [35]. Joint recurrence plots are the Hadamard product of the recurrence plots of the considered sub-systems. For two systems x and y, the JRP is defined by the relation:

(17) $$JR_{ij}=\Theta \left(\varepsilon_{x}-\|\vec{x_{i}}-\vec{x_{j}}\|\right)\times \Theta \left(\varepsilon_{y}-\|\vec{y_{i}}-\vec{y_{j}}\|\right),\;\;\;i,j,=1,\ldots ,N$$

JRP compare the simultaneous occurrence of recurrences in two (or more) systems, and therefore can be used to detect their correlation in the phase space. JRPs have also been used for estimation of the direction of coupling between interacting systems [36].

As an RP is characterized by typical patterns, the typology and texture of these structures can be used for understanding the underlying dynamics of the time series [37]. The most common features in an RP are single isolated points, vertical, horizontal and diagonal lines. Single isolated points correspond to states with a rare occurrence, which do not persist over time and are characterized by high fluctuations. Vertical and horizontal lines correspond to states which do not change significantly during a certain period of time. Diagonal lines occur when the trajectory visit the same region at different times and a segment of the trajectory runs parallel to another segment. The length of a diagonal is proportional to the duration of such a process. Therefore, in JRP, long diagonal structures correspond to a similar time evolution of the two processes.

However, graphical analysis is limited, and new insights can be obtained by means of the so-called recurrence quantification analysis (RQA) [31]. RQA is based on several indicators, which are based on the distributions of diagonal, vertical and horizontal lines. Of particular relevance for the subject of this paper is the average diagonal length (ADL):

(18) $$ADL=\frac{\sum_{l=l_{min}}^{N}lP\left(l\right)}{\sum_{l=l_{min}}^{N}P\left(l\right)}$$

where P(l) is the frequency distribution of the lengths l of the diagonal lines.

JRP are an efficient tool for analyzing the causal relation of short time series (see, e.g., [38,39]).

### 2.4. Causal Decomposition (CD)

The CD method [21] is based on the instantaneous phase dependency between cause and effect. It uses Empirical Mode Decomposition (EMD) [40,41] to break down the time series into a finite number of intrinsic mode functions (IMF), which are time-varying single-frequency functions. The set of IMFs forms a complete and nearly orthogonal basis for the original signal. IMFs are characterized by two distinctive properties: the numbers of extrema and of zero crossings are equal, or differ by one at most, and the mean envelope determined by the upper and lower envelopes is zero.

EMD is a fully adaptive data-driven method that does not make any prior assumptions about the linearity or stationarity of the signal; this generality represents its main advantage. The main drawback resides in the mode mixing: sometimes a signal at a certain scale is located in different IMFs or an IMF may incorporate signals at rather different scales. The ensemble mode decomposition (EEMD) [42] was introduced in order to cope with this problem. The decomposition is performed on data with several white noise realizations. The added noise has the role collating the portion of the signal of comparable scale in one IMF [21]. The level of added noise should minimize the IMF’s pairwise correlation, while maintaining an acceptable level of non-orthogonal leakages [40].

In the end, the ensemble mean is used to represent the final result, with the different scales being separated in a natural way. However, EEMD is not fully reversible, which means that the sum of all IMFs reconstructs the signal only up to a residual.

After decomposing the two time series, the Hilbert transform [43] can be used to calculate the instantaneous phase of each IMF. Then the phase coherence between corresponding IMFs in the two time series is evaluated. The instantaneous phase coherence can be defined as:

(19) $$Coh\left(S_{1j},S_{2j}\right)=\frac{1}{T}\int_{0}^{T}exp\left(i\Delta \Phi_{12j}\left(t\right)dt\right)$$

where $S_{1j}$ and $S_{2j}$ are the j-th IMF component of the first and second time series, respectively, and ${\Delta \Phi }_{12j}$ is the instantaneous phase difference.

The main idea of the CD method is to successively remove one IMF component from the signal corresponding to the driven time series and to re-decompose it into a new set of IMFs. If the removed IMF is causally related to the driver time series, then the redistribution of the phase dynamic will be related only to the dynamic of the driven time series. The phase coherence between the pair of IMFs in the two time series will be reduced. This variation can be measured by means of the variance-weighted Euclidean distance between the phase coherence of the pair of IMFs decomposed from the original and re-decomposed signals [21]:

(20) $$D\left(S_{1j}\to S_{2j}\right)=\sqrt{\overset{m}{\underset{j=1}{\sum }}W_{j}{\left[Coh\left(S_{1j},S_{2j}\right)-Coh\left(S_{1j},S_{2j}^{\prime }\right)\right]}^{2}}\;D\left(S_{2j}\to S_{1j}\right)=\sqrt{\overset{m}{\underset{j=1}{\sum }}W_{j}{\left[Coh\left(S_{1j},S_{2j}\right)-Coh\left(S_{1j}^{\prime },S_{2j}\right)\right]}^{2}}\;W_{j}=\left(Var_{1j}\cdot Var_{2j}\right)/\overset{m}{\underset{j=1}{\sum }}\left(Var_{1j}\cdot Var_{2j}\right)$$

where the prime symbol indicates the IMF removal followed by re-decomposition. D∈(0,1) is interpreted as the level of absolute causal strength. The relative causal strength between IMF $S_{1j}$ and $S_{2j}$ can be quantified by the relation:

(21) $$\begin{array}{c} C\left(S_{1j}\to S_{2j}\right)=\;D\left(S_{1j}\to S_{2j}\right)/\left[D\left(S_{1j}\to S_{2j}\right)+D\left(S_{2j}\to S_{1j}\right)\right]\\ C\left(S_{2j}\to S_{1j}\right)=\;D\left(S_{2j}\to S_{1j}\right)/\left[D\left(S_{1j}\to S_{2j}\right)+D\left(S_{2j}\to S_{1j}\right)\right] \end{array}$$

If the ratio is close to 0.5, it means that there is no causal relationship or there is equal causal strength. Otherwise it is an indication that a significant causal influence exists.

### 2.5. Complex Networks (CN)

A recently developed measure for the characterization of interconnected dynamical systems coupling [22], based on the transformation of time series in a complex network, can also be used for studying the interlink of malaria occurrence and various climatic variables. The method is based on the cross-visibility (CV) concept [44], which extends the use of the visibility graphs (VG) [45] for the study of coupled time series. VGs are based on the representation of time series by vertical bars, which create a landscape of peaks and valleys. For every bar in the time series, a node is created in the complex network. Two nodes are connected if the corresponding bars are reciprocally visible in the landscape. For two time series $\left\{x_{i}\right\}$ and $\left\{y_{i}\right\}$, the CV method creates the links of the complex network by connecting the components of $\left\{x_{i}\right\}$ if they are reciprocally visible through the obstacles created by the shifted time series $\left\{y_{k}\right\}$ = {$y_{k}$ − $y_{i}$ + $x_{i}$} [44]. The connection rules are given by the relations:

(22) $$y_{k}\leq y_{i}+\frac{x_{j}-x_{i}}{j-i}\left(k-i\right),\;\;\;\;i<\forall k<j$$

or

(23) $$y_{k}\geq y_{i}+\frac{x_{j}-x_{i}}{j-i}\left(k-i\right),\;\;\;\;i<\forall k<j$$

As shown in [22], the weighted adjacency matrix (WAM) is defined by the relation:

(24) $$a_{ij}^{w}=\{\begin{array}{c} \;dist\left(y_{i}-y_{j}\right),\;if\;Eq.\;\left(18\right)\;or\;Eq.\;\left(19\right)\;is\;satified\\ 0,\;\;\;otherwise \end{array}$$

where $dist\left(y_{i}-y_{j}\right)$ is the metric distance. An alternative solution, used in this paper, is to use a similarity measure between the time series segments located in between the connected nodes for weighting the connections. The distances based on $L_{p}$ norms:

(25) $$d_{L_{p}}{\left(x,y\right)}_{\left[i↔j\right]}={\left(\overset{j}{\underset{k=i}{\sum }}{\left(x_{k}-y_{k}\right)}^{p}\right)}^{\frac{1}{p}}$$

represent a straightforward solution (see, e.g., [46,47]). In particular, the Euclidean distance, which is de facto the most applied similarity metric, has been chosen for the present study.

WAM can be used for monitoring the complexity of the network, which is linked with the degree of coupling between time series. WAM is a 2D matrix and it can be therefore represented as an image. The image entropy [48] is defined as

(26) $$p_{i}=\frac{H\left(i\right)}{\sum_{i}H\left(i\right)},\;\;\;\;i=1,\ldots ,N_{H}$$

where H is the histogram of pixel intensities in the image, H(i) is the number of pixels with a certain intensity and $N_{H}$ the number of intensity bins in the image.

The entropy has a lower value for images with repeating patterns or a gradual change of grey-levels, while high values are reached for images with pixel values changing randomly. For usual images, the areas characterized by a smooth changing of grey levels, the existence of blocks of uniform pixel values or the presence of repeating patterns of texture will have lower values than those for which the pixel values change rapidly or in a random way.

For independent evolving time series, the reciprocal visibility between two bars (corresponding to two components of the time series) is frequently limited by the obstacles created by the other time series. When coupled, the time series lean towards synchronization, and the WAM image evolves into a less random structure, characterized by lower entropy. The degree of coupling between the coupled time series can be therefore evaluated by means of the image entropy. The coupling measure is defined by the relation:

(27) Q=−H(CVN)

where the minus sign has been introduced in order to have an increase of Q with the coupling strength, as most of the existent coupling measures. The image entropy has proved to be a good indicator of the changes in the complex network structure. However, future work will be dedicated to searching for more refined measures based on various intrinsic characteristics of the network [49].

## 3. Results and Discussion

### 3.1. Malaria Data

The methods described in the previous section were used to analyse the data reported by Haque et al. [8] (HAQUE-data) and Hanf et al. [9] (HANF-data). The former reference was chosen because it contains a detailed study of the relationship between malaria epidemics and a comprehensive series of climatic factors: rainfall, temperature, humidity, sea surface temperature (SST), El Niño Southern Oscillation (ENSO) and NDVI. The latter reference was selected because it reports the investigation of a weak relation between ENSO and the malaria epidemic, and therefore it can be viewed as a test for the sensitivity of the proposed methods. For the data in [8], the Niño Region 3 (NINO-3) index was used to monitor the ENSO variations. The Niño-n (n = 1, 2, 3, 3.4, 4) indices correspond to regions crossed by different ships’ tracks, enabling the historic records of ENSO. NINO-3 captures ENSO anomalies in the (5° S–5° N and 150° W–90° W) region. This region was once the primary focus for monitoring and predicting El Niño and La Niña events. Its values are available at the NOAA ESRL Physical Sciences Division Data [50]. In [9], ENSO is quantified by means of the Southern Oscillation Index (SOI). The SOI index is based on the observed sea level pressure differences between Tahiti and Darwin, in Australia (West Pacific). The smoothed time series of the SOI correspond very well with changes in ocean temperatures across the eastern tropical Pacific; prolonged periods of negative/positive SOI values coincide with abnormally warm/cold ocean waters across the eastern tropical Pacific typical of El Niño/La Niña episodes. This information is available at the Australian Bureau of Meteorology [51]. The NDVI index was prepared by Haque et al. based on the data library of the International Research Institute (IRI) of Lamont Doherty Earth Observatory (LDEO) at Columbia University, USA [8]. The other climatological data have been retrieved from the local meteorological stations [8,9].

Haque et al. collected the malaria cases related to the Rangamati district hospital (Bangladeshi Highlands, 22° 40′ N, 92° 11′ E) from January 1989 to December 2008. Hanf et al. considered the clinical malaria episodes diagnosed for patients consulting the emergency services of Cayenne hospital, French Guyana, between 1996 and 2009. More details about the malaria data collection can be found in [8,9]. There are some limitations concerning these data. The first is related to underrepresentation; only the cases reported by the hospital emergency service consultation were taken into account. Also, the data is restricted to a limited geographical area. Therefore, the conclusions cannot be easily extrapolated. However, this is beyond the scope of this paper, which aims only at showing the potential of the presented methods, as an alternative set of tools, for the investigation of malaria–climate interlink.

**Figure 1:**
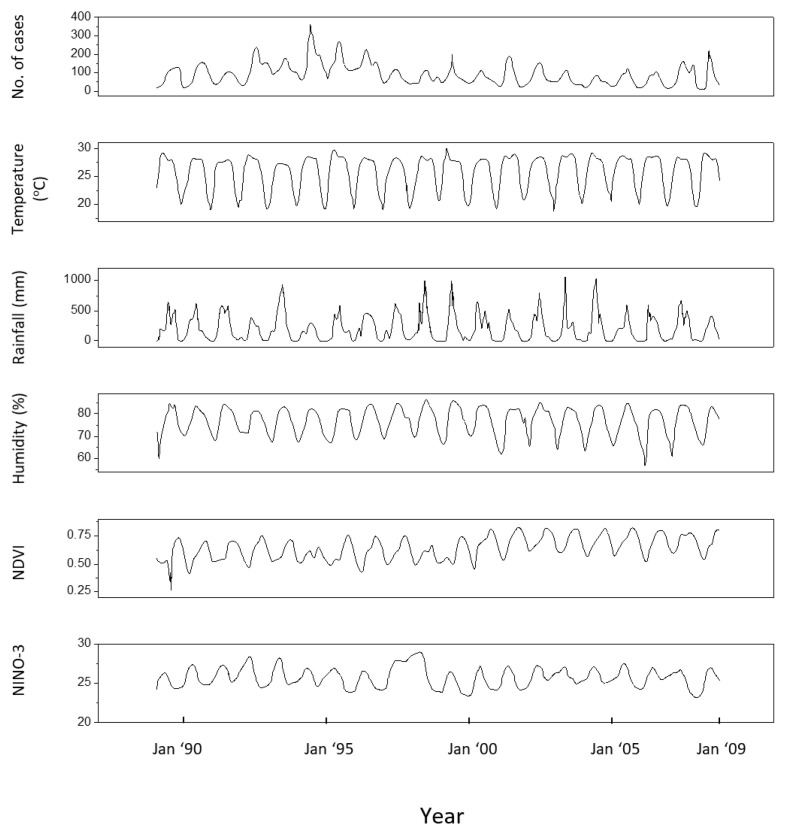
Time series corresponding to Rangamati, 1989–2008 [8]. From top bottom: number of malaria cases, temperature, rainfall, humidity, NDVI and NINO-3.

**Figure 2:**
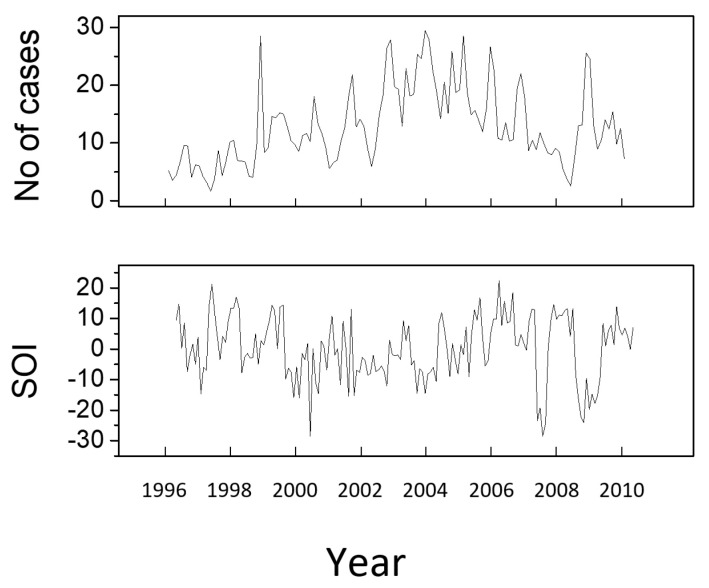
Time series corresponding to the malaria cases at the Cayenne General Hospital and to SOI for the time interval 1996–2009 [9].

All malaria and climatic data used in this paper are presented in Figure 1 and Figure 2. The interlink between the number of malaria cases and the other factors considered was studied in [8,9] using a time lag of a maximum of 3 months between the corresponding time series. In the present approach, the maximum time lag is extended to 6 months.

### 3.2. Data Analysis

The malaria data was analysed by means of the five methods described above. The KGC method has been applied by using a second-degree order polynomial kernel. In the case of the TE method, the size of the candidate terms was set as L=10. Following [25], τ was chosen for each time series by taking the first value in the range (1÷10), which leads to a value of the autocorrelation of the series below 1/e. The typical length of the conditioning vectors is in the range (4÷6). The number of quantization levels has been chosen to be Q = 6. For the recurrence plots we considered an embedding dimension m = 2, while the threshold of the recurrence plots has been set to ε = 0.25, which represents approximatively 10% of the mean phase space diameter. In the case of the CD method, 300 noise realizations with a level r of 0.25 standard deviations of the time series were used for the EEMD decomposition.

**Figure 3:**
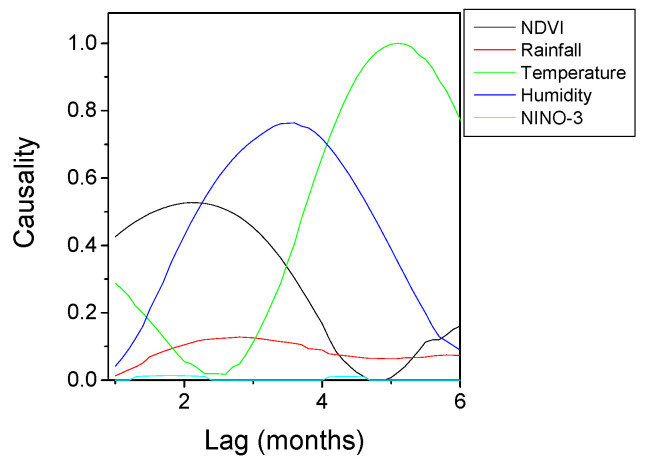
KGC causality index variation with the time lag for the time series pairs constructed with the evolution of the number of malaria cases, on the one hand, and several factors (NDVI, Rainfall, Temperature, Humidity, El Niño) on the other hand. The curves are normalized to their common maximum value.

**Figure 4:**
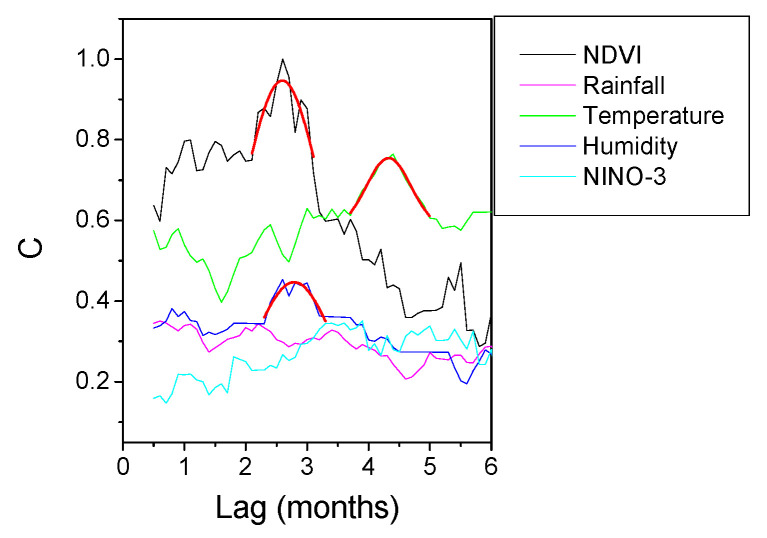
TE causality index variation with the time lag for the time series pairs constructed with the evolution of the El Niño, on the one hand, and several physical indicators (NDVI, Rainfall, Temperature, Humidity) on the other hand. The curves are normalized to their common maximum value.

**Figure 5:**
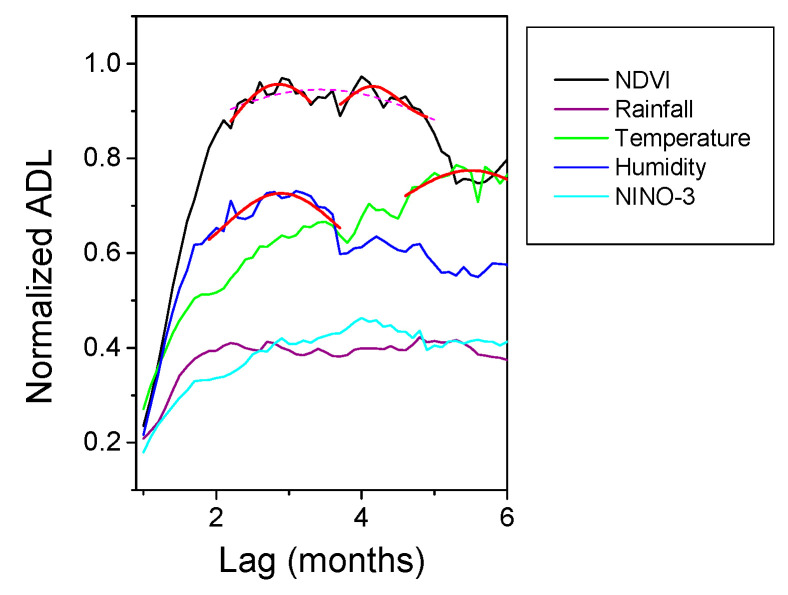
Evolution of JRP-ADL indicator with the time lag for the time series pairs constructed with the evolution of the number of malaria cases, on the one hand, and several factors (NDVI, Rainfall, Temperature, Humidity, El Niño) on the other hand. The curves are normalized to their common maximum value.

**Figure 6:**
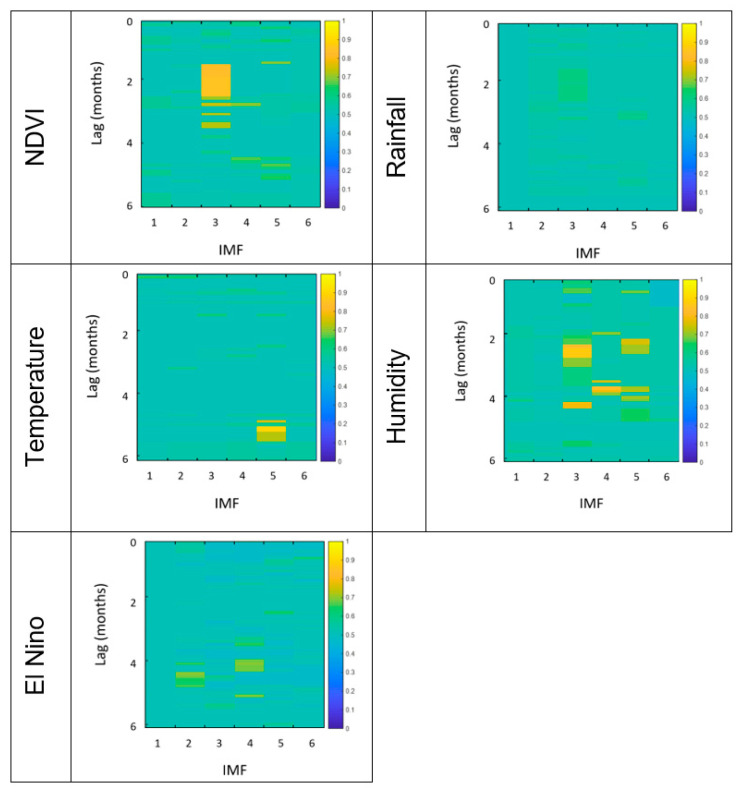
Representation of the relative causal strength for different time lags for the time series pairs constructed with the evolution of the number of malaria cases, on the one hand, and several factors (NDVI, Rainfall, Temperature, Humidity, El Niño) on the other hand.

**Figure 7:**
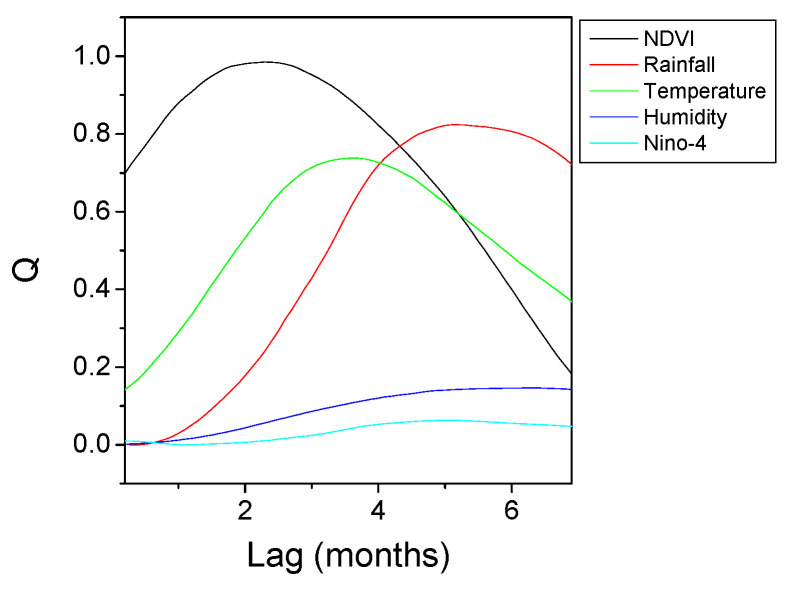
Evolution of the complex network complexity measure Q with the time lag for the time series pairs constructed with the evolution of the number of malaria cases, on the one hand, and several factors (NDVI, Rainfall, Temperature, Humidity, El Niño) on the other hand. The curves are normalized to their common maximum value.

**Figure 8:**
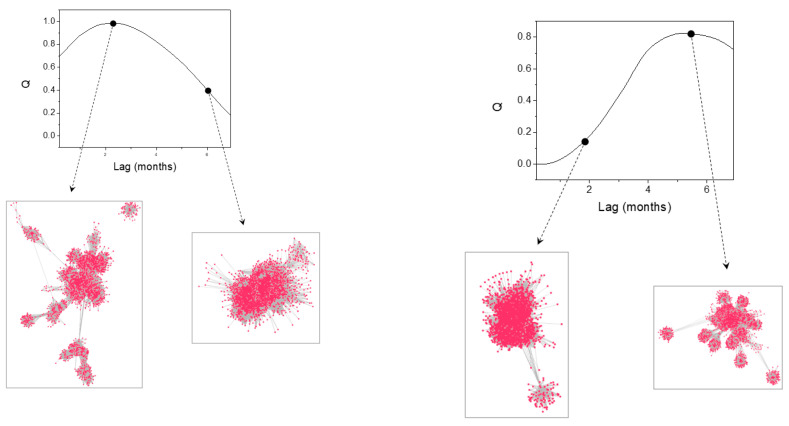
Illustration of the complex network structure evolution with the increased coupling of the time series for the pairs constructed with the number of malaria cases, on the one hand, and NDVI (**left**) and temperature time series (**right**). For the time lag corresponding to maximal causal influence the networks develop several distinct clusters. The networks have been created using the ‘prefuse’ force directed lay-out in Cytoscape 3.7.1 [52].

The results of HAQUE-data analysis are presented in Figure 3, Figure 4, Figure 5, Figure 6 and Figure 7. Figure 3 and Figure 4 depict the evolution of the causality index (Equations (10) and (13)) vs. the time lag for the KGC and TE methods, respectively. The ADL indicator (Equation (18)), characterizing the RPs obtained for different lags, is presented in Figure 5. The map of the relative causal strength (Equation (21)) is shown in Figure 6. This map is presented as a 6 x 60 pixel image; each line contains 6 values quantifying the relative causal strength corresponding to 6 IMFs; each line refers to a different value of the time lag. Finally, Figure 7 show the evolution of the coupling measure given by Equation (27) vs. the time lag for the CN method. Figure 8 illustrates the structure of the complex network for the time lag corresponding to a maximal coupling and for a lower one. For an increased coupling, the network develops a clearly coherent structure, characterized by several clusters.

The positions of the peaks in these graphs are listed in Table 1. For the CD method, the last column of the table lists the limits of the time intervals corresponding to the bright regions in the maps, as a ‘peak’ is difficult to define for this method.

The results show a dependence of the number of malaria cases on the evolution of NDVI, temperature and humidity. The five methods provide coherent values of the time lags at which the dependence reaches a maximum. The estimates are in agreement with those reported in [8], where the causality was investigated for a maximum time lag of three months, and in which an association between the number of malaria cases and the NDVI index was revealed. For the CD method, a second peak appears at a time lag in the interval 3.8–4.2 months, but this is not confirmed by the other methods. A link with the rainfall and ENSO seems to be non-existent. The CD methods show a weak dependence on the NINO-3 index but, again, this is not confirmed by the other methods.

With regard to the strength of the causal influence, it is difficult to directly compare the values given by the different methods. The values presented in Figure 3, Figure 4, Figure 5, Figure 6 and Figure 7 are all normalized to the maximum value, but the range of variation before normalization is specific for each method. However, a ranking of the causal influence can be established for each method. These ranks are listed in Table 2, where, for the three climatic parameters that seem to have an influence on the number of malaria cases, the strongest influence is denoted by the value 1 and the weakest by the value 3. The table shows that TE, RP, CD and CN methods give causal interlinks of the same order.

**Figure 9:**
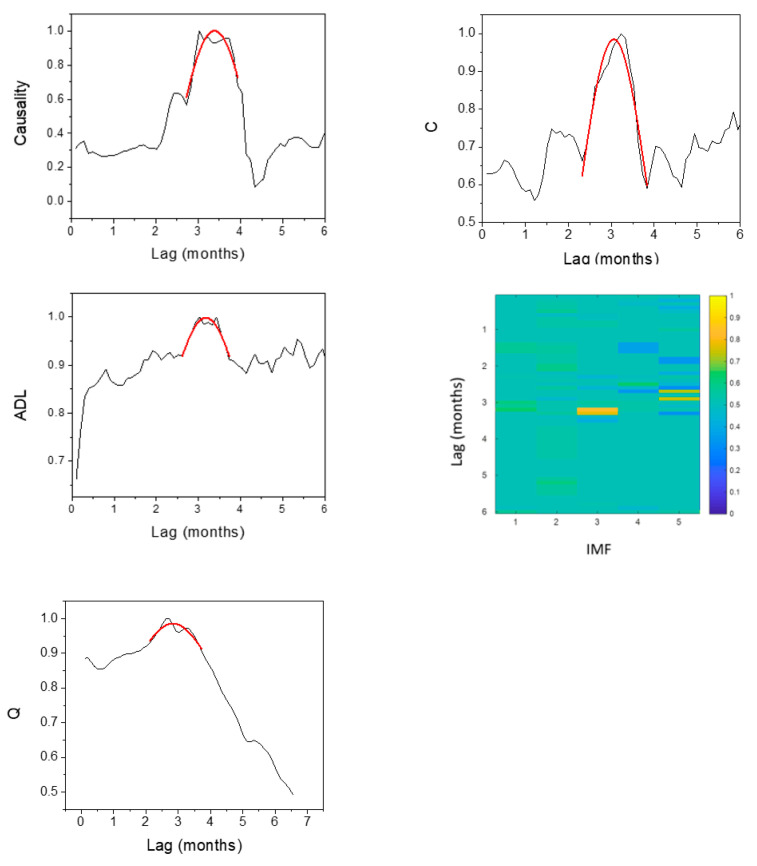
Evolution of the causality indicators versus the time lag: KGC causality index (top-left), TE causality index (top-right) and JRP-ADL indicator (middle-left), CD relative causal strength (middle-right) and CN coupling measure (bottom), with the time lag for the time series pairs constructed with the evolution of the number of malaria cases, on one hand, and El Niño index, on the other hand.

As already mentioned, the HANF-data was included in this analysis because of the weak causal influence of ENSO on the number of malaria cases. The study reported in [9] states that ENSO is responsible for 4% of the variation of the number of cases. Despite this weak interlink, the KGC, TE, RP and CN methods are able to reveal it clearly (see Figure 9). The CD maps also reveal a feature for approximatively the same time lag value; however, the feature existence is less certain as it extends over very small time intervals. The time lag values of the maximum causal intervals determined with each method are listed in Table 3.

## 4. Conclusions

Malaria is one of the major health problems in the world, and therefore, forecasting its occurrence would be extremely beneficial. However, forecasting requires a good preliminary understanding of the relations between malaria and various factors. As a vector-borne disease, malaria’s occurrence is strongly related to climatic phenomena through complex processes. As the methods especially developed for identifying causal relations between time series have rarely been used in malaria occurrence studies, in this paper, a series of five methods were applied for the study of two cases reported in the literature. The techniques succeed in providing coherent results for the time lag between malaria occurrence and various climatic factors. This may help in quantifying the uncertainty in the estimates; indeed, the small discrepancies between the estimates of the various methods can be interpreted as the confidence intervals in the results. The methods are also able to identify causation correctly even in the case of a weak coupling between the dynamical processes.

However, as various detailed studies have shown [53,54], the detection of the coupling delay of dynamical systems is a very difficult problem, far from being completely solved. The performances of the causality detection methods varied with the type of the studied dynamical systems. In some cases, the methods worked well only if the strength of the coupling exceeded a certain threshold. Sometimes they even provided contradictory results. The existence of a strong oscillatory component, quite common for climatic data, could also be a factor deteriorating the quality of the estimations. Therefore, blind application could lead to erroneous results. A multi-path approach, based on the application of several methods based on different principles, was explicitly adopted in the present work to at least partly remedy these difficulties and to increase the confidence in the results. With appropriate caution, we believe that the methods described in this paper will be more widely used in the future to investigate the causal links between malaria and the factor influencing its diffusion.

## Figures and Tables

**Table 1:** Time lag values (months) corresponding to a maximal causal influence of several climatic factors (NDVI, rainfall, temperature, humidity, El Niño) on the number of malaria cases.

	Method				
Parameter	Kernel Granger Causality	Transfer Entropy	Recurrence Plots	Causal Decomposition	Complex Networks
NDVI	2.2	2.6	2.8, 4.2	[1.7–2.5]	2.3
Rainfall	-	-	-	-	-
Temperature	5.1	4.4	5.5	[4.8–5.5]	5.4
Humidity	3.4	2.8	2.9	[2.1–2.7] [3.8–4.2]	3.6
NINO-3	-	-	-	[4.0–4.4]	-

**Table 2:** Ranking of the causal influence of different climatic parameters.

	Method				
Parameter	Kernel Granger Causality	Transfer Entropy	Recurrence Plots	Causal Decomposition	Complex Networks
NDVI	3	1	1	1	1
Temperature	1	2	2	2	2
Humidity	2	3	3	3	3

**Table 3:** Time lag values corresponding to the maximal causal influence of El Nino on the number of malaria cases.

Method	LAG (Months)
Kernel Granger causality	3.35
Transfer Entropy	3.06
Recurrence plots	3.18
Causal decomposition	[3.1–3.3]
Complex networks	2.83

## References

[1] World Health Organization (WHO) and WHO Global Malaria Programme. http://www.who.int/malaria/about_us/en/index.html,.

[2] Lindsay S.W., Martens W.J. (1998). Malaria in the African highlands: past, present and future. Bull. WHO.

[3] Lafferty K.D. (2009). The ecology of climate change and infectious diseases. Ecology.

[4] Pascual M., Bouma M.J. (2009). Do rising temperatures matter. Ecology.

[5] Field C.B., Barros V.R., Dokken D.J., Mach K.J., Mastrandrea M.D., Bilir T.E., Chatterjee M., Ebi K.L., Estrada Y.O., Genova R.C. (2014). Climate change 2014: Impacts, adaptation, and vulnerability. Part A: global and sectoral aspects. Contribution of working group II to the fifth assessment report of the intergovernmental panel on climate change. IPCC (2014) Summary for Policymakers.

[6] Carter R., Mendis K.N. (2002). Evolutionary and historical aspects of the burden of malaria. Clin. Microbiol. Rev..

[7] Eikenberry S.E., Gumel A.B. (2018). Mathematical modeling of climate change and malaria transmission dynamics: A historical review. J. Math. Biol..

[8] Haque U., Hashizume M., Glass G.E., Dewan A.M., Overgaard H.J., Yamamoto T. (2010). The Role of Climate Variability in the Spread of Malaria in Bangladeshi Highlands. PLoS ONE.

[9] Hanf M., Adenis A., Nacher M., Carme B. (2011). The role of El Niño southern oscillation (ENSO) on variations of monthly Plasmodium falciparum malaria cases at the cayenne general hospital, 1996–2009, French Guiana. Malar. J..

[10] Sadoine M.L., Smargiassi A., Ridde V., Tusting L.S., Zinszer K. (2018). The associations between malaria, interventions, and the environment: A systematic review and meta-analysis. Malar. J..

[11] Mabaso M.L.H., Ndlovu N.C. (2012). Critical review of research literature on climate-driven malaria epidemics in sub-Saharan Africa. Public Health.

[12] Guo C., Yang L., Ou C.-Q., Li L., Zhuang Y., Yang J., Zhou Y.-X., Qian J., Chen P.-Y., Liu Q.-Y. (2015). Malaria incidence from 2005–2013 and its associations with meteorological factors in Guangdong, China. Malar. J..

[13] Akpalu W., Codjoe S.N.A. (2013). Economic Analysis of Climate Variability Impact on Malaria Prevalence: The Case of Ghana. Sustainability.

[14] Hwang S.-M., Yoon S.-J., Jung Y.-M., Kwon G.-Y., Jo S.-N., Jang E.-J., Kwon M.-O. (2016). Assessing the impact of meteorological factors on malaria patients in demilitarized zones in Republic of Korea. Infect. Dis. Poverty.

[15] Granger C.W.J. (1969). Investigating Causal Relations by Econometric Models and Cross-spectral Methods. Econometrica.

[16] Marinazzo D., Pellicoro M., Stramaglia S. (2008). Kernel Method for Nonlinear Granger Causality. Phys. Rev. Lett..

[17] Marinazzo D., Pellicoro M., Stramaglia S. (2008). Kernel-Granger Causality and the Analysis of Dynamical Networks. Phys. Rev. E.

[18] Schreiber T. (2000). Measuring Information Transfer. Phys. Rev. Lett..

[19] Faes L., Nollo G., Porta A. (2011). Information-based detection of nonlinear Granger causality in multivariate processes via a nonuniform embedding technique. Phys. Rev. E.

[20] Eckmann J.P., Kamphorst S.O., Ruelle D. (1987). Recurrence plot of dynamical systems. Europhys. Lett..

[21] Yang A.C., Peng C.-K., Huang N.E. (2018). Causal decomposition in the mutual causation system. Nat. Commun..

[22] Craciunescu T., Murari A., Gelfusa M. (2018). Improving Entropy Estimates of Complex Network Topology for the Characterization of Coupling in Dynamical Systems. Entropy.

[23] Wiener N. (1956). The Theory of Prediction.

[24] Barnett L., Barrett A.B., Seth A.K. (2009). Granger causality and transfer entropy are equivalent for Gaussian variables. Phys. Rev. Lett..

[25] Faes L., Nollo G., Porta A. (2011). Information domain approach to the investigation of cardio-vascular, cardiopulmonary and vasculo-pulmonary causal couplings. Front. Physiol..

[26] Small M. (2005). Applied Nonlinear Time Series Analysis: Applications in Physics, Physiology and Finance.

[27] Porta A., Baselli G., Liberati D., Montano N., Cogliati C., Gnecchi-Ruscone T., Malliani A., Cerutti S. (1998). Measuring regularity by means of a corrected conditional entropy in sympathetic outflow. Biol. Cybern..

[28] Marwan N., Romano M.C., Thiel M., Kurths J. (2007). Recurrence plots for the analysis of complex systems. Phys. Rep..

[29] Craciunescu T., Murari A. (2016). Geodesic distance on Gaussian Manifolds for the robust identification of chaotic systems. Nonlinear Dyn..

[30] Mindlin G.M., Gilmore R. (1992). Topological analysis and synthesis of chaotic time series. Phys. D.

[31] Zbilut J.P., Webber C.L. (1992). Embeddings and delays as derived from quantification of recurrence plots. Phys. Lett. A.

[32] Kennel M.B., Brown R., Abarbanel H.D.I. (1992). Determining embedding dimension for phase-space reconstruction using a geometrical construction. Phys. Rev. A.

[33] Thiel M., Romano M.C., Kurths J. (2004). How much information is contained in a recurrence plot?. Phys. Lett. A.

[34] Donner R.V., Zou Y., Donges J.F., Marwan N., Kurths J. (2010). Recurrence networks—a novel paradigm for nonlinear time series analysis. New J. Phys..

[35] Marwan N. (2008). A historical review of recurrence plots. Eur. Phys. J. Spec. Top..

[36] Romano M.C., Thiel M., Kurths J., Grebogi C. (2007). Estimation of the direction of the coupling by conditional probabilities of recurrence. Phys. Rev. E.

[37] Mocenni C., Facchini A., Vicino A. (2011). Comparison of recurrence quantification methods for the analysis of temporal and spatial chaos. Math. Comput. Model..

[38] Murari A., Craciunescu T., Peluso E., Lerche E., Gelfusa M., Contributors J. (2017). On efficiency and interpretation of sawteeth pacing with on-axis ICRH modulation in JET. Nucl. Fusion.

[39] Murari A., Craciunescu T., Peluso E., Gelfusa M., Contributors J. (2017). Detection of causal relations in time series affected by noise in tokamaks using geodesic distance on gaussian manifolds. Entropy.

[40] Huang N.E., Shen Z., Long S.R., Wu M.C., Shih H.H., Zheng Q., Yen N.-C., Tung C.C., Liu H.H. (1998). The empirical mode decomposition and the Hilbert spectrum for nonlinear and non-stationary time series analysis. Proc. Math. Phys. Eng. Sci..

[41] Wu Z., Huang N.E., Long S.R., Peng C.-K. (2007). On the trend, detrending, and variability of nonlinear and nonstationary time series. Proc. Natl. Acad. Sci. USA.

[42] Wu Z.H., Huang N.E. (2008). Ensemble empirical mode decomposition: A noise assisted data analysis method. Adv. Adapt. Data Anal..

[43] Hahn S.L., Poularikas A.D. (1996). Hilbert transforms. The Transforms and Applications Handbook.

[44] Mehraban S., Shirazi A.H., Zamani M., Jafari G.R. (2016). Coupling between time series: A network view. EPL.

[45] Lacasa L., Luque B., Ballesteros F., Luque J., Nun J.C. (2008). From time series to complex networks: The visibility graph. PNAS.

[46] Ding H., Trajcevski G., Scheuermann P., Wang X., Keogh E. (2008). Querying and Mining of Time Series Data: Experimental Comparison of Representations and Distance Measures. Proc. VLDB Endow..

[47] Iglesias F., Kastner W. (2013). Analysis of Similarity Measures in Times Series Clustering for the Discovery of Building Energy Patterns. Energies.

[48] Silva L.E.V., Senra Filho A.C.S., Fazan V.P.S., Felipe J.C., Murta Junior L.O. (2016). Two-dimensional sample entropy: Assessing image texture through irregularity. Biomed. Phys. Eng. Express.

[49] Rak R., Kwapień J., Oświęcimka P., Zięba P., Drożdż S. (2019). Universal features of mountain ridge networks on Earth. J. Complex Netw..

[50] NOAA ESRL Physical Sciences Division Data. https://www.esrl.noaa.gov/psd/data/gridded/index.html.

[51] S.O.I. (Southern Oscillation Index) Archives. http://www.bom.gov.au/climate/glossary/soi.shtml.

[52] Shannon P., Markiel A., Ozier O., Baliga N.S., Wang J.T., Ramage D., Amin N., Schwikowski B., Ideker T. (2003). Cytoscape: A software environment for integrated models of biomolecular interaction networks. Genome Res..

[53] Coufal D., Jakubík J., Jajcay N., Hlinka J., Krakovská A., Paluš M. (2017). Detection of coupling delay: A problem not yet solved. Chaos.

[54] Krakovská A., Jakubík J., Chvosteková M., Coufal D., Jajcay N., Paluš M. (2018). Comparison of six methods for the detection of causality in a bivariate time series. Phys. Rev. E.

